# First person – Anneliese Ceisel and Gianna Graziano

**DOI:** 10.1242/dmm.053120

**Published:** 2026-07-24

## Abstract

First Person is a series of interviews with the first authors of a selection of papers published in Disease Models & Mechanisms, helping researchers promote themselves alongside their papers. Anneliese Ceisel and Gianna Graziano are co-first authors on ‘ Cell death analysis of inducible, titratable, neurodegenerative disease models in zebrafish and human stem cell-derived retinal organoids’, published in DMM. Anneliese is a PhD candidate in the lab of Jeff Mumm at Johns Hopkins School of Medicine, Baltimore, MD, USA, investigating regenerative medicine. Gianna is Research Specialist II in the same lab, investigating regeneration in zebrafish.

**Figure:**
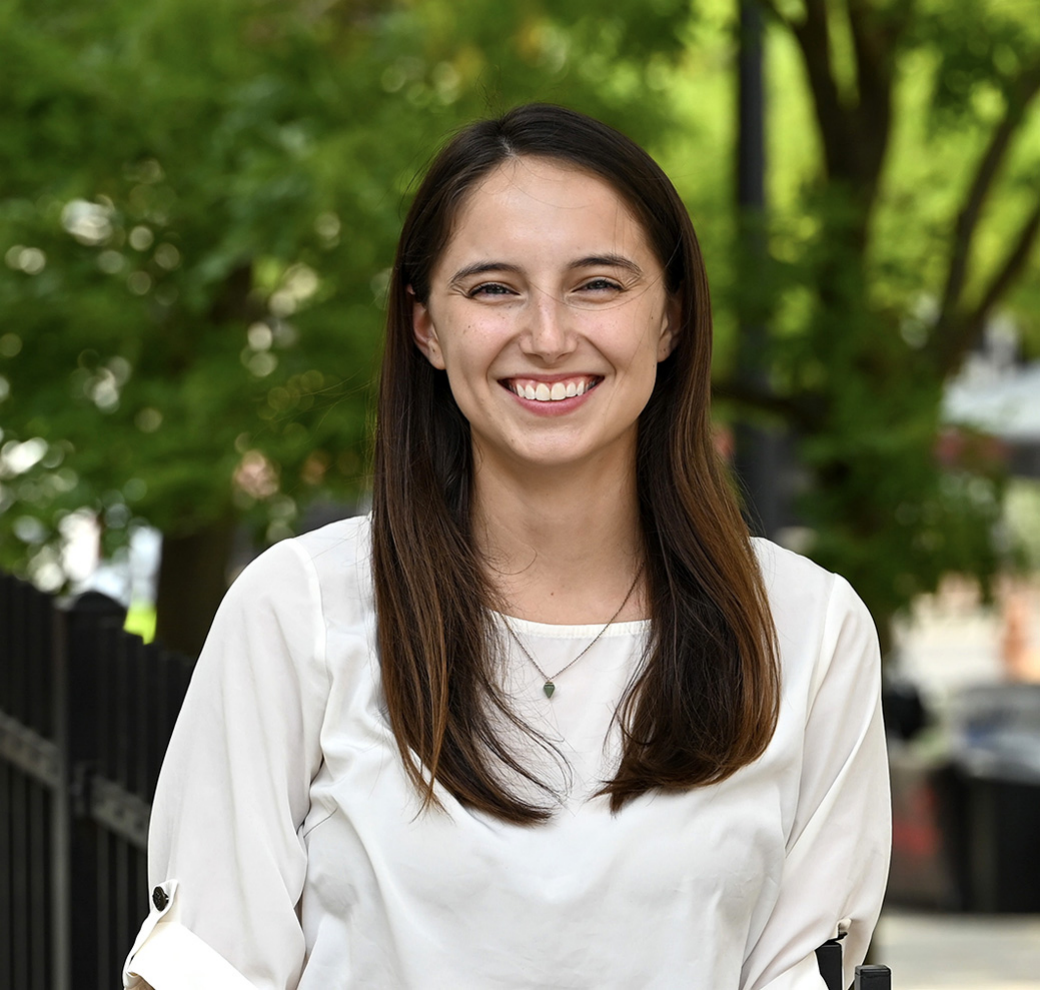
Anneliese Ceisel

**Who or what inspired you to become a scientist?**

**A.C.:** My dad was a part-time beekeeper growing up, and every year I would help out with honey harvesting. As a child, spinning down the honey in a hand-cranked centrifuge was always my favourite part, and I think being a part of that honey-harvesting process started my initial interest in biology. It wasn't until high school though that I became interested in the intersection of biology and medicine. I was learning about advances in biology and, specifically, induced pluripotent stem cells. I saw how cells and scaffolds were being used for tissue generation and engineering, such as the Vacanti mouse. I was really excited about the potential for regrowing new tissues and organs, and was really excited by the work from pioneers in the field like Dr Anthony Atala. I was hearing news stories from companies like Miromatrix who were engineering new ways to make organs compatible for transplant. And I knew I wanted to be a part of bringing those opportunities to patients.

**G.G.:** As a kid, I spent a lot of time outside, wondering how everything around me worked. When I got to high school and took freshman-year biology, so many of the questions I'd always had floating around my brain were finally answered, but I just kept wanting to know more. By the time I was finishing up my undergraduate degree in biological sciences and figuring out how I wanted to spend the rest of my life, I knew that I would be bored in any other field but research. Simply, I became a scientist because it's so exciting to learn how life functions, especially on the cellular and molecular scale; as a scientist, I not only get to spend every day learning, but I also get to share that newfound knowledge with the world.

**What is the main question or challenge in disease biology you are addressing in this paper? How did you go about investigating your question or challenge?**

**A.C./G.G.:** In disease biology, it's common to generate tools to model the onset and progression of a disease. In our case, we were interested in modelling neurodegeneration in zebrafish and retinal organoids but wanted to leverage genetic tools that allow control over the onset and extent of neuron loss. To make our neurodegeneration models more disease relevant, we wanted to understand the mechanism(s) of cell death elicited by our models to see if it aligns with previous reports of the mechanism(s) of cell death in various neurodegenerative diseases. In fish, we used chemical and genetic inhibition of key components of different cell death pathways to see whether the inhibition of any of these pathways would lead to an increase in neuron survival upon induction of neurodegeneration. If so, this would suggest that the pathways identified play an important role in the cell death elicited by our system. We extended this model into retinal organoids and assessed the pathways involved by quantifying key proteins in those pathways during cell death.

We found that cells in our model die by parthanatos, a type of cell death that is common in diseases like retinitis pigmentosa and Parkinson's disease, where cell loss plays a large role in disease progression

**How would you explain the main findings of your paper to non-scientific family and friends?**

**A.C./G.G.:** We have a model of cell loss that we use in fish and human lab-grown mini-tissues to study neurodegenerative disease and their potential treatments. We wanted to understand how apt our model is, so we looked to see if the cells were dying in the same way as they do in human disease. We found that cells in our model die by parthanatos, a type of cell death that is common in diseases like retinitis pigmentosa and Parkinson's disease, where cell loss plays a large role in disease progression. This suggests that our model could be useful for mimicking these diseases in particular.

**What are the potential implications of these results for disease biology and the possible impact on patients?**

**A.C./G.G.:** Our results demonstrate that this disease model can be relevant to different neurodegenerative diseases and is adaptable to different model organisms. This is incredibly useful as this particular model is quick and easy to utilize, and allows control over the onset and duration of the neurodegeneration. This can (and did) facilitate large-scale screens to identify therapeutics, bringing patients more, and potentially better, options for treatment.

**Figure:**
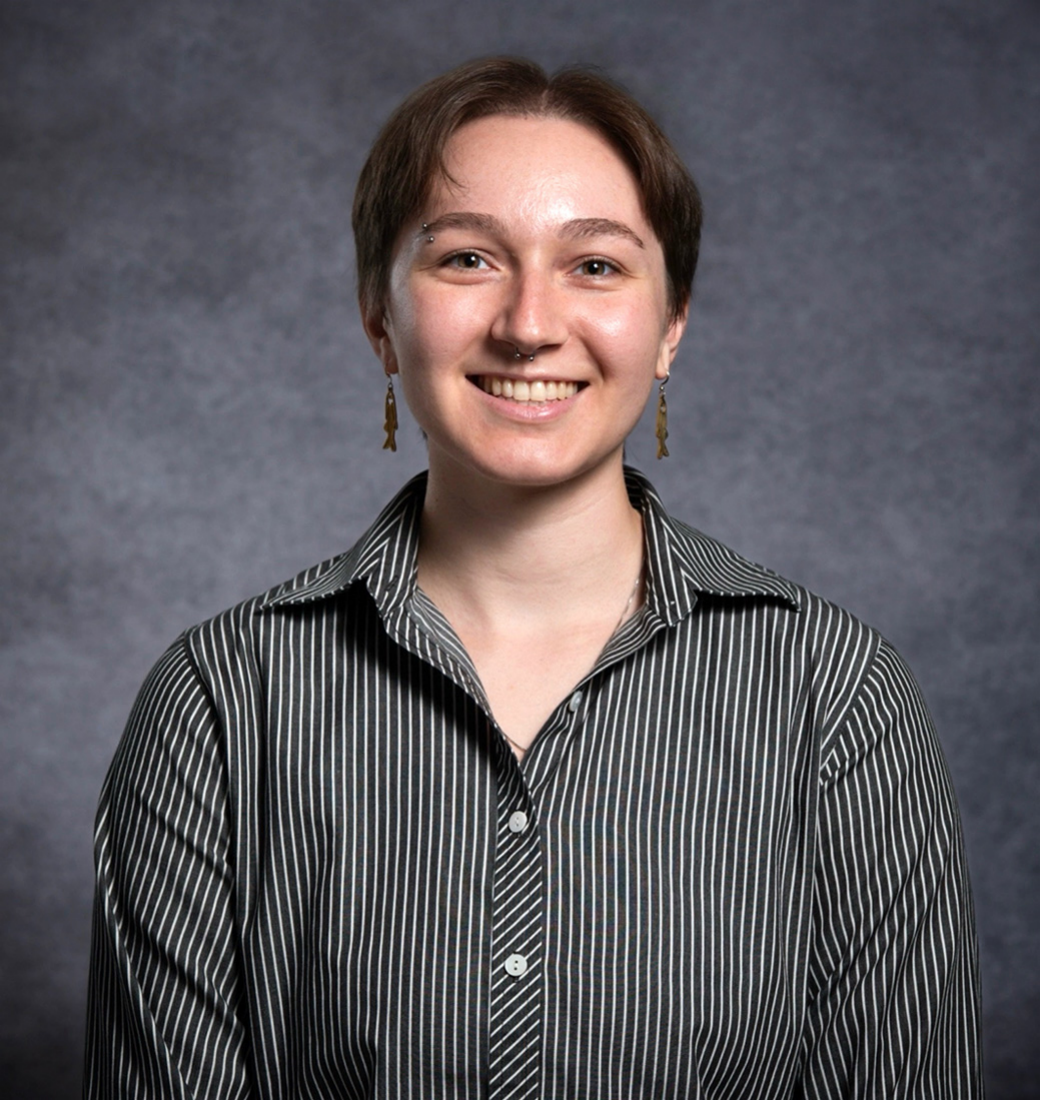
Gianna Graziano

**Figure:**
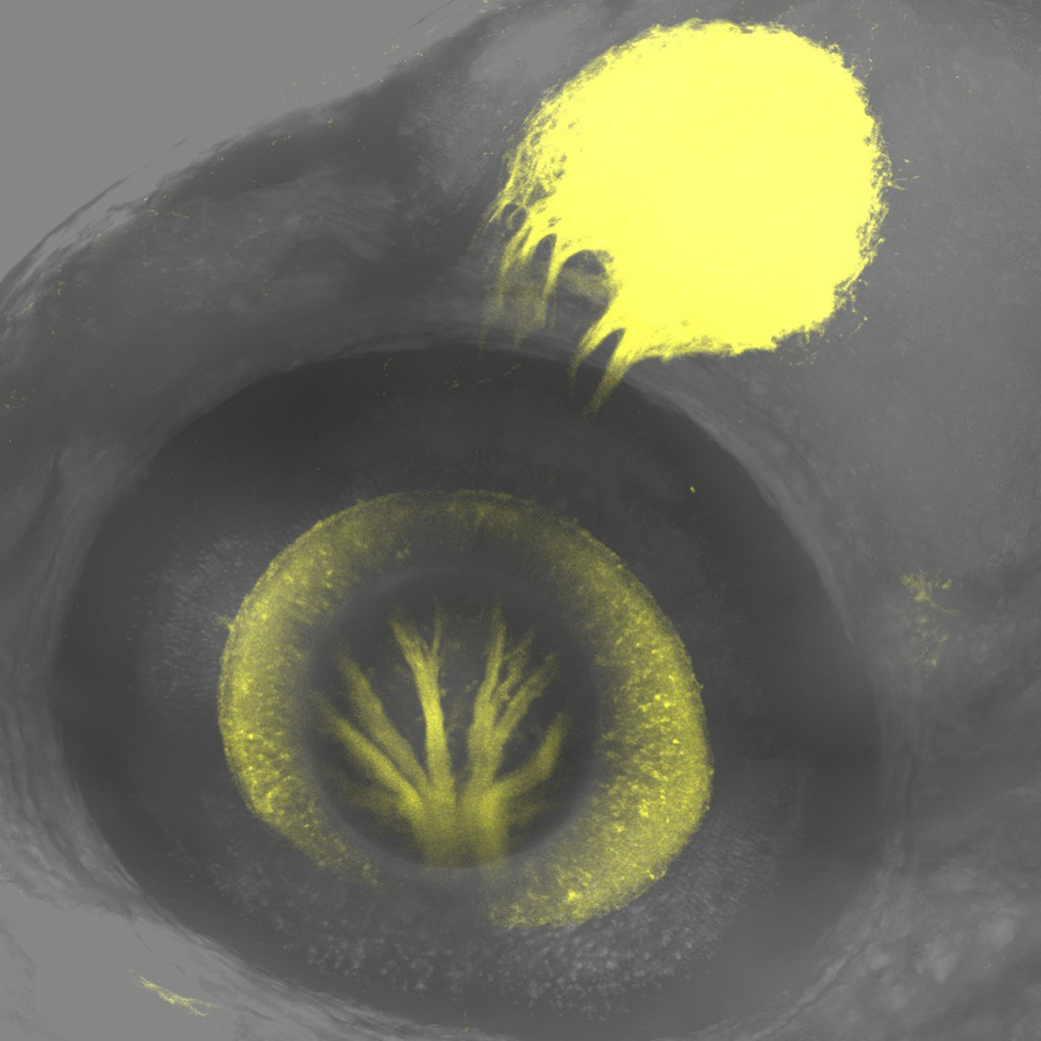
YFP-labelled retinal ganglion cells in the retina and optic tectum of a 5-day-old live zebrafish.

**Why did you choose DMM for your paper?**

**A.C./G.G.:** We understand the goal of the journal to be interdisciplinary in scope, cover a wide array of diseases and provide insight into treatments of human disease. The model we use is similarly applicable to a variety of neurodegenerative diseases, and the goal is to use it to identify chemical or genetic therapeutics that can slow down, stop or rescue cell loss during disease. As we are studying the mechanisms with which nitroreductase/metronidazole-induced cell-loss models function, we chose DMM for our paper because we thought it would be the most fitting. We also appreciate that the journal is peer reviewed and open access.

… building in more work–life balance and finding ways to minimize the publish-or-perish culture would be helpful not just for PIs, but for the students who are doing a lot of the benchwork to bring those papers and projects into reality

**Given your current role, what challenges do you face and what changes could improve the professional lives of other scientists in this role?**

**A.C.:** One of the biggest challenges I have faced is probably burnout. It's easy to pile on interesting projects and necessary tasks as a student, and there are so many other roles and responsibilities I take on in the lab. When something goes wrong or takes longer than expected, it can push you back and challenge you even more. I know I am not alone in this. I think when people get burnt out, they are more likely to make mistakes, get sloppy with their work or just stop caring. If we want science to be more robust, I think building in more work–life balance and finding ways to minimize the publish-or-perish culture would be helpful not just for PIs, but for the students who are doing a lot of the benchwork to bring those papers and projects into reality.

**G.G.:** As a Research Specialist, many of the challenges I face revolve around time management and dedicating my full focus and effort to many different projects simultaneously. I have independent projects, but my role also requires that I aid graduate students and postdocs with their work, mentor undergrads, maintain fish facilities and lines, generate resources for the lab and for collaborators, prepare manuscripts (as exemplified here), among other things. It can be hard to juggle all of this without getting overwhelmed. Fortunately, in my lab, research technologists and specialists are treated fairly, given a lot of independence and shown respect for their work. However, I know that this is not the case for a lot of techs – the scientific community could really benefit from encouraging techs to pursue their own research interests and design their own projects, rather than working them to the bone and limiting them to monotonous tasks that the rest of the lab just doesn't want to do. This job is often a way that young scientists gain experience and start figuring out what they want to do before taking further steps in their career, and we should start treating the people in this role accordingly; if we don't, we're closing ourselves off to a lot of ideas/perspectives we wouldn't have otherwise.

**What's next for you?**

**A.C.:** As I approach my PhD thesis defence, I am looking into translational research opportunities at different biotech companies. I am hoping to further my career in the field of regenerative medicine and tissue engineering and, ultimately, help patients in need.

**G.G.:** I am starting my PhD in Biochemistry, Cellular, and Molecular Biology here at Hopkins at the end of August. I'm sad about leaving the Mumm lab after being here for over 4 years, but I'm so excited to see where grad school takes me next!

**Tell us something interesting about yourself that wouldn't be on your CV**

**A.C.:** In grade school, I attended a Spanish dual-language program where half of my classes were taught fully in Spanish. As such, I learned math and science in Spanish until I was 11. Nowadays, I continue to use and practise my Spanish, including by teaching science lessons in English and Spanish to kids!

**G.G.:** Outside of the lab, one of my main hobbies is cycling. I use a lot of my vacation days on bikepacking trips (like backpacking, but on a bike instead of your feet). I've cycled and camped my way across the states of Maryland, Pennsylvania, Missouri, Ohio and New York. One day, I hope to ride across the entire USA!

## References

[DMM053120C1] Ceisel, A., Graziano, G., Emmerich, K., Shi, X., Kam, T.-I., Flores-Bellver, M., Vergara, M. N., Aparicio-Domingo, S., Brenerman, B. M., Vielle, A. et al. (2026). Cell death analysis of inducible, titratable, neurodegenerative disease models in zebrafish and human stem cell-derived retinal organoids. *Dis. Model. Mech.* 19, dmm052747. 10.1242/dmm.052747 42497345 PMC13474575

